# B Cell Functions Can Be Modulated by Antimicrobial Peptides in Rainbow Trout *Oncorhynchus mykiss*: Novel Insights into the Innate Nature of B Cells in Fish

**DOI:** 10.3389/fimmu.2017.00388

**Published:** 2017-04-04

**Authors:** Xu-Jie Zhang, Peng Wang, Nu Zhang, Dan-Dan Chen, Pin Nie, Jia-Le Li, Yong-An Zhang

**Affiliations:** ^1^College of Fisheries and Life Science, Shanghai Ocean University, Shanghai, China; ^2^State Key Laboratory of Freshwater Ecology and Biotechnology, Institute of Hydrobiology, Chinese Academy of Sciences, Wuhan, China; ^3^University of Chinese Academy of Sciences, Beijing, China

**Keywords:** antimicrobial peptides, phagocytosis-enhancing activity, B cells, innate nature, rainbow trout *Oncorhynchus mykiss*

## Abstract

B cells in fish were recently proven to have potent innate immune activities like macrophages. This inspired us to further explore the innate nature of B cells in fish. Moreover, antimicrobial peptides (AMPs) are representative molecules of innate immunity, and they can modulate the functions of macrophages. These make fish an appropriate model to study the interactions between B cells and AMPs. Interestingly, the results in this study revealed that the IgM^+^ and IgT^+^ B cells of rainbow trout could express multiple AMP genes, including four cathelicidin genes and one β-defensin gene. The expression levels of the cathelicidin genes were obviously higher than that of the β-defensin gene. Further studies revealed that intracellular, extracellular, *in vitro*, and *in vivo* stimulations could significantly increase the expression of the cathelicidin genes in trout IgM^+^ and IgT^+^ B cells but not the expression of the β-defensin gene, indicating that cathelicidin peptides are the main innate immune effectors of trout B cells. More interestingly, we found that cathelicidin peptides could significantly enhance the phagocytic, intracellular bactericidal, and reactive oxygen species activities of trout IgM^+^ and IgT^+^ B cells, a phenomenon previously reported only in macrophages, and these activities might also be mediated by the P2X_7_ receptor. These results collectively suggest that B cells play multiple roles in the innate immunity of fish, and they provide new evidence for understanding the close relationship between B cells and macrophages in vertebrates.

## Introduction

The immune system of fish is composed of an innate immune system and an adaptive immune system. Unlike the higher vertebrates, fish live in an aquatic environment and depend more on their innate immune system to protect themselves from various pathogenic microbes (1). Phagocytosis, one of the innate immune responses, plays important roles in the defense against the invasion of pathogenic bacteria, as well as in the initiation of the adaptive immune responses (2, 3). In mammals, the phagocytosis of pathogenic bacteria is mainly conducted by professional phagocytes, such as monocytes, macrophages, and granulocytes (4). Unlike these phagocytes, early studies have proven that primary B cells in mammals lack phagocytic capability (5, 6). However, recent studies demonstrated that B cells in fish have potent *in vitro* and *in vivo* phagocytic abilities like macrophages (7–10). After phagocytosis, fish B cells can form “phagolysosomes” to kill the internalized bacteria, and they further act as antigen-presenting cells to present antigens recovered from the phagocytosed bacteria to CD4^+^ T cells to initiate the adaptive immune responses (7, 9). In amphibians (*Xenopus laevis*) and reptiles (*Trachemys scripta*), a proportion of B cells is also phagocytic, but the activities are significantly lower than those of fish B cells (7, 11). A recent study also revealed that viruses can induce the early activation of fish B cells toward an antigen-presenting profile (12). The abovementioned reports indicate that B cells in ectotherms, especially in fish, still play multiple roles in the innate immunity.

Antimicrobial peptides (AMPs) represent an evolutionarily old component of the innate immune system in all organisms (13, 14). They form the first line of host defense against infectious microorganisms prior to stimulating animals’ adaptive immune systems (13–15). Among them, cathelicidins, defensins, and liver-expressed antimicrobial peptides (LEAPs) represent the major and well-studied AMP families in animals (14, 16, 17). These AMPs exhibit a broad spectrum of antimicrobial activities against bacteria, viruses, fungi, and even some parasites (14, 15, 17). In mammals, AMPs are mainly produced by epithelial cells and neutrophils (18, 19). Moreover, human monocytes/macrophages and lymphocytes—including NK, T, and B cells—can also produce the cathelicidin peptide LL-37 and the α-defensins HNP 1–3 (18). Like T and B cells in humans, mouse T and B cells can also produce the cathelicidin peptide mCRAMP (20). In addition, human B cells have been proven to produce β-defensin 2 (21). Previous studies have shown that human NK and T cells have direct bactericidal activities, and the AMPs produced by these cells are candidate effectors for this activity (18). Recent studies have revealed that B cells in fish also possess potent direct bactericidal activities (7, 8), but the effector molecules that mediate this activity have not been characterized. This inspires us to further explore whether AMPs are produced by fish B cells and are effectors for their bactericidal activity.

Most AMPs are constitutively expressed in various tissues, where their expression can also be induced during infection, with their expression activating more complex immune responses in addition to their direct antimicrobial activities (22). Human α-defensins are found to be potent chemoattractants for macrophages (23). Human cathelicidin peptide LL-37 can enhance the phagocytic, intracellular bactericidal, reactive oxygen species (ROS), and lysosome formation activities of macrophages (24, 25). A β-defensin in Atlantic cod (*Gadus morhua*) can stimulate the phagocytic activity of attached head kidney leukocytes (HKLs) (26). As mentioned above, fish B cells possess potent innate immune activities like macrophages. Thus, we have reasons to speculate that fish AMPs can act on B cells, like human AMPs acting on macrophages. The results of this study will confirm this hypothesis.

Over the past decade, researchers have achieved considerable progress in the immune functions of fish B cells (7–9, 12, 27). To date, two B cell lineages have been identified in rainbow trout (*Oncorhynchus mykiss*), including IgM^+^ and IgT^+^ B cells, which mainly participate in systemic and mucosal immunity, respectively (8, 27, 28). Moreover, several AMP genes have been identified in rainbow trout, including the cathelicidin genes (CATH-1a, CATH-1d, CATH-2a, and CATH-2b) (29), the β-defensin genes (DB-1, DB-2, DB-3, and DB-4) (30), and the LEAP genes [LEAP-1 (hepcidin), LEAP-2A, and LEAP-2B] (31, 32). The special living environment and evolutionary status of rainbow trout make it an interesting model to study the modulatory roles of AMPs on B cells. This not only facilitates a fully understanding of the innate immune functions of B cells in fish but also provides new insights into the evolution of B cells in vertebrates.

## Materials and Methods

### Fish

Rainbow trout (100–150 g) obtained from Zhanghe Reservoir Rainbow Trout Farm (Jingmen, China) were maintained and acclimated to the laboratory conditions as previously described (29).

### Cell Sorting

Trout leukocytes from peripheral blood leukocytes (PBLs) and HKLs were obtained with 51/34% discontinuous Percoll (GE Healthcare) density gradients as previously described (33). Flow cytometric sorting of trout IgM^+^ and IgT^+^ B cells were performed as described previously (8). Briefly, PBLs or HKLs were double stained with mouse anti-trout IgM and anti-trout IgT monoclonal antibodies (mAbs) (1 µg/ml each) at 4°C for 30 min. In parallel, an irrelevant mouse anti-HA Ab was used as a negative control. APC-goat anti-mouse IgG1 (Jackson Scientific) and PE-goat anti-mouse IgG2b (Jackson Scientific) were used as secondary Abs to detect the IgM^+^ and IgT^+^ B cells, respectively. Lymphocytes and myeloid leukocytes in trout PBLs and HKLs were gated, and the IgM^+^, IgT^+^, and IgM^−^IgT^−^ (double negative, DN) lymphocytes as well as myeloid leukocytes were sorted by fluorescence-activated cell sorting (FACS) using BD FACSAria™ III (BD Biosciences). In some experiments, in order to maximize cell viability, magnetic-activated cell sorting (MACS) of trout IgM^+^ and IgT^+^ B cells was performed as previously described (9). Briefly, PBLs or HKLs were blocked with 5% fetal bovine serum (Gibco) at 4°C for 15 min, then cell suspensions were stained with mouse anti-trout IgM or anti-trout IgT mAb (1 µg/ml each) at 4°C for 30 min, washed with MACS buffer (2 mM EDTA and 0.5% BSA in PBS), and incubated at 4°C for 15 min with anti-mouse IgG magnetic beads (Miltenyi Biotec). The IgM^+^ and IgT^+^ B cells were sorted with LS separation columns (Miltenyi Biotec), according to the manufacturer’s instructions. The purities of the MACS-sorted IgM^+^ and IgT^+^ B cells were further determined by flow cytometry as previously described (9), and the purity was >93 and >91% for IgM^+^ and IgT^+^ B cells, respectively (Figure S1 in Supplementary Material).

### Total RNA Isolation and cDNA Synthesis

Total RNA from about 6 × 10^5^ sorted cells/sample was isolated by using the RNeasy Mini Kit (Qiagen) and treated with RNase-Free DNase (Qiagen) to digest genomic DNA according to the manufacturer’s instructions. First-strand cDNA was synthesized from the total RNA with SuperScript First-Strand Synthesis System (Invitrogen) and stored at −20°C for use.

### Expression of AMP Genes in Normal Trout B Cells

Expression patterns of AMP genes in the sorted IgM^+^, IgT^+^, and DN lymphocytes as well as myeloid leukocytes from peripheral blood and head kidney of healthy trout were performed by RT-PCR as described previously (8). Primer sets specific for trout AMP genes and the reference gene elongation factor 1a (EF-1a) were shown in Table 1. All PCRs were performed in 20-µl reaction volume containing 14 µl water, 2 µl 10× buffer, 2 µl dNTP (2.5 mM each), 0.25 µl Ex Taq HS DNA Polymerase (TaKaRa), 1 µl primer set (10 µM each), and 0.75 µl cDNA. The amplification program was as follows: 95°C for 5 min, followed by 46 cycles of 95°C for 20 s, 60°C for 20 s, and 72°C for 30 s, and then 72°C for 5 min. Amplified DNA was analyzed using 1.5% (w/v) agarose gel electrophoresis stained with ethidium bromide and visualized with a UV transilluminator (Bio-Rad). The expression levels of AMP genes in the sorted IgM^+^, IgT^+^, and DN lymphocytes as well as myeloid leukocytes were further analyzed by quantitative real-time PCR (qPCR) using the specific primer sets (Table 1) in a CFX real-time PCR detection system (Bio-Rad) as previously described (29). Amplification efficiencies of all the primer sets were between 95 and 105%, calculated by using twofold series dilution of cDNA in qPCR. Specificity of the primer sets was verified by the dissociation curves and sequencing the qPCR products (data not shown). The relative expression levels of trout AMP genes were determined by the cycle threshold (Ct) method and normalized against the internal control EF-1a using the 2^−ΔCt^ method (34).

**Table 1:** **Primers used for gene cloning and expression**.

Primer	Sequences (5′–3′)	Target gene	Application
CATH-1F	AGAAGAAGCAAAGTCAGAATATG	CATH-1a/1d	RT-PCR
CATH-1R	TATGCAAAGCGATTTCCATCAC		
CATH-2F	GAAGAGGCAAGGACAGCGGA	CATH-2a/2b	RT-PCR
CATH-2R	AGACAAAAGAGTTACCAGAAGCA		
DB-1F	GGTTTTCCTATTGCTTAATGTTGTGG	DB-1	RT-PCR
DB-1R	GACACACAGTTAAGTCATGG		
DB-2F	GCTGACAGCAGTGCAAGCTGATGACAC	DB-2	RT-PCR
DB-2R	GCAAAGCACAGCATCTTAATCTGC		
DB-3F	GCTTGTGGAATACAAGAGTCATCTGC	DB-3	RT-PCR and quantitative real-time PCR (qPCR)
DB-3R	GCATACATTCGGCCATGTACATCC		
DB-4F	GCAACTCTTCTAAAGAACAGT	DB-4	RT-PCR
DB-4R	CGTGGGCGACACAGCATACAAATCC		
LEAP-1F	TGCAGTGGTGGTCGTCCTCG	LEAP-1	RT-PCR
LEAP-1R	GACGCTTGAACCTGAAATGCTCC		
LEAP-2aF	CTGGTGCTCGGTCCGCAGGCGT	LEAP-2A	RT-PCR
LEAP-2aR	CCCCTGCAAATCCCAGTGGAGC		
LEAP-2bF	TGCACGGTCAAAACCACAGCA	LEAP-2B	RT-PCR
LEAP-2bR	AGGAATGAACTGCCCCACTGA		
qCATH-1aF	AGGCAAGCAACAACCTGAACAC	CATH-1a	qPCR
qCATH-1aR	CCCCAAGACGAGAGACACAAT		
qCATH-2F	CAACACCCTCAACACTGACCG	CATH-2a/2b	qPCR
qCATH-2R	GAATCTTTTCTACCCATCTTAGG		
qEF-1aF	CAAGGATATCCGTCGTGGCA	EF-1a	RT-PCR and qPCR
qEF-1aR	ACAGCGAAACGACCAAGAGG		
P2X_7_R-F	ATGCCTTGTAAACTGCTAAATCTA	P2X_7_R	cDNA amplification
P2X_7_R-R	GCTGGGGTACTCCTCTCTGA		
qP2X_7_R-F	CCCAAATACTCCTTCCGCCG	P2X_7_R	qPCR
qP2X_7_R-R	TTCCTGCCTTTCCAAACACCAT		

### Immunofluorescence Staining

Immunofluorescence was performed to detect the expression of cathelicidin peptide CATH-2a in trout B cells as previously described with minor modifications (29, 35). Briefly, MACS-sorted IgM^+^ and IgT^+^ B cells from peripheral blood and head kidney of trout were dript on the surface of a piece of slide glass and stained with rabbit anti-trout CATH-2a Ab (29) overnight at 4°C. In parallel, the irrelevant rabbit anti-GST Ab was used as negative control. After washing, the cells were stained for 1.5 h at room temperature with Alexa Fluor 488-AffiniPure donkey anti-rabbit IgG (Jackson Scientific) (2.5 µg/ml) as secondary Ab to detect trout CATH-2a in trout B cells. After washing, the cells were stained with DAPI (Beyotime), and the images were acquired using a confocal microscope (Zeiss).

### Expression of AMP Genes in Phagocytic and Non-Phagocytic Trout B Cells

Phagocytosis by trout IgM^+^ and IgT^+^ B cells was performed as described previously (8). Briefly, fluorescent beads (Fluoresbrite Yellow Green Microspheres, 1.0 µm in diameter; Polysciences) in 300 μl L-15 medium (Sigma-Aldrich) were seeded in 24-well plates (Nunc) at a density of 10^7^ beads/well and pelleted by centrifugation at 2,500 *g* for 5 min. Then trout PBLs or HKLs in 300 μl L-15 medium were added to each well at a cell:bead ratio of 1:10, followed by incubation for 3 h at 17°C. After incubation, cell suspensions were centrifuged (100 *g* for 10 min at 4°C) over a cushion of 3% (weight/volume) BSA (Thermo Scientific) in PBS supplemented with 4.5% d-glucose (Sigma-Aldrich) to remove the non-ingested beads. The collected cells were stained with anti-trout IgM and anti-trout IgT mAbs as described above, followed by FACS to sort the phagocytic and non-phagocytic IgM^+^ and IgT^+^ B cells using BD FACSAria III (BD Biosciences). Cells were collected and subjected to total RNA isolation and cDNA synthesis as described above. The relative expression levels of AMP genes in the phagocytic and non-phagocytic trout B cells were determined by the Ct method and normalized against the internal control EF-1a using the 2^−ΔΔCt^ method (34).

### Stimulation of Trout B Cells with LPS and *Aeromonas salmonicida*

MACS-sorted IgM^+^ and IgT^+^ B cells [6 × 10^5^ cells/100 µl of medium/well in a 96-well microplate (Nunc)] from peripheral blood and head kidney of healthy trout were incubated with PBS or 25 µg/ml LPS (*Escherichia coli* 0111:B4; Sigma-Aldrich) or heat-killed pathogenic *A. salmonicida* at a cell:bacterium ratio of 1:10 in L-15 medium for 8 h at 17°C. For *A. salmonicida* stimulation, bacteria were heat inactivated at 65°C for 1 h, washed and pelleted by centrifugation at 2,800 *g* at 4°C for 5 min prior to incubation with trout B cells. After incubation, the stimulated cells were collected, and then subjected to total RNA isolation and cDNA synthesis as described above. The relative expression levels of trout AMP genes in the IgM^+^ and IgT^+^ B cells under normal and challenged situations were further analyzed by qPCR using the primer sets and conditions as described above.

### Infection of Trout with *A. salmonicida*

Healthy trout were injected intraperitoneally with PBS or *A. salmonicida* (2 × 10^7^ CFU/ml in PBS, 100 µl/fish) as previously described (36). The IgM^+^ and IgT^+^ B cells were MACS sorted from trout peripheral blood and head kidney at 30 h postinfection, and then subjected to total RNA isolation and cDNA synthesis as described above. The relative expression levels of AMP genes in the IgM^+^ and IgT^+^ B cells from healthy and infected trout were further analyzed by qPCR using the primer sets and conditions as described above.

### Phagocytosis Assay

Phagocytic activity of trout B cells stimulated with cathelicidin peptides was measured as previously described (24, 37) with some modifications. Briefly, PBLs in 100 μl L-15 medium were seeded in 96-well plates (Nunc) at a cell density of 2 × 10^5^ cells/well and incubated for 3 h at 17°C with trout CATH-1a or CATH-2a at a final concentration of 2 µM. Cathelicidin peptides used in this study were synthesized as previously described (29). Non-stimulation controls were included, with PBS instead of peptide. After incubation, cells were harvested and added to the wells of a new plate for 1 h at 17°C, which were previously plated with fluorescent beads (Fluoresbrite Yellow Green Microspheres, 1.0 µm in diameter; Polysciences) by centrifugation at 2,500 *g* for 5 min at a cell:bead ratio of 1:15. After incubation, cell suspensions were centrifuged (100 *g* for 10 min at 4°C) over a cushion of 3% (weight/volume) BSA (Thermo Scientific) in PBS supplemented with 4.5% d-glucose (Sigma-Aldrich) to remove the non-ingested beads. The collected cells were stained with anti-trout IgM and anti-trout IgT mAbs as described above, followed by flow cytometric analysis using BD FACSVerseTM (BD Biosciences). Phagocytic activity is expressed as the percentage of cells that ingested beads.

### Intracellular Bactericidal Assay

Intracellular bactericidal activity of trout B cells stimulated with cathelicidin peptides was measured as previously described (7, 25) with some modifications. Briefly, log-phase *E. coli* ATCC25922 in 300 μl L-15 medium (Sigma-Aldrich) were seeded in 24-well plates (Nunc) at a density of 10^7^ CFU/well, pelleted by centrifugation at 2,500 *g* for 5 min, then 10^6^ trout PBLs or HKLs in 300 μl L-15 medium were added to each well, and incubated for 4 h at 17°C. After incubation, cell suspensions were centrifuged (100 *g* for 10 min at 4°C) over a cushion of 3% (weight/volume) BSA (Thermo Scientific) in PBS supplemented with 4.5% d-glucose (Sigma-Aldrich) to remove the non-ingested bacteria. Pelleted cells were resuspended in L-15 medium supplemented with 50 µg/ml gentamicin (Amresco) and incubated for 1 h at 17°C to kill the non-internalized bacteria. Then IgM^+^ and IgT^+^ B cells were MACS sorted as described above. Sorted cells (2 × 10^5^ cells/100 µl of L-15 medium/well) were seeded in a 96-well plate (Nunc) and further incubated with trout CATH-1a or CATH-2a at a final concentration of 2 µM for 3 h at 17°C. Non-stimulation controls were included, with PBS instead of peptide. After incubation, cells were washed three times with PBS (Invitrogen), then lysed with sterilized water and vortexed for 30 s. Lysates were serially diluted, plated onto LB agar plates, and incubated at 37°C overnight. Colonies of viable bacteria were counted, and results are presented as percentage of the colony number for stimulation group/control group.

### Cellular ROS Activity

The level of ROS in trout B cells stimulated with cathelicidin peptides was measured with a 2,7-dichlorofluorescin diacetate (DCFH-DA) cellular ROS assay kit (Nanjing Jiancheng Bioengineering Institute) as previously described (25) with some modifications. Briefly, trout PBLs and HKLs were incubated with *E. coli* ATCC25922 for 4 h at 17°C, and then the IgM^+^ and IgT^+^ B cells were MACS sorted as described in Section “Intracellular Bactericidal Assay.” Sorted cells [6 × 10^5^ cells/100 μl L-15 medium/well in a 96-well microplate (Nunc)] were incubated for 3 h at 17°C with trout CATH-1a or CATH-2a at a final concentration of 2 µM. Non-stimulation controls were included, with PBS instead of peptide. After incubation, cells were stained with 100 µl diluted DCFH-DA for 1 h at 17°C in the dark. The fluorescence intensity of the cells was read using a fluorescence microplate reader (SpectraMax M5, Molecular Devices), with an excitation wavelength of 485 nm and an emission wavelength of 538 nm. Relative ROS activity was calculated as percentage of the value for stimulation well/control well.

### Cloning and Expression of P2X_7_ Receptor (P2X_7_R) in Trout B Cells

Using the reported sequence of zebrafish (*Danio rerio*) P2X_7_R (GenBank accession no. NP_945335.1) to blast against GenBank database at the National Center for Biotechnology Information,^1^ a predicted trout P2X_7_R gene was obtained (GenBank accession no. CDQ65050.1). Gene-specific primers P2X_7_R-F and P2X_7_R-R (Table 1) were used to amplify the transcript of trout P2X_7_R, and the PCR product was sequenced with an ABI3730XL sequencer (Applied Biosystems) by Tsingke Company (Wuhan, China), and the sequence was submitted to GenBank^2^ under the accession number KY088056. In order to ensure the correctness of the cloned sequence, the phylogenetic relationship of trout P2X_7_R with selected vertebrate P2X receptor family sequences was constructed based on the amino acid sequence alignment using the MEGA 4.1 package, with a neighbor-joining algorithm and 1,000 bootstrap replications (Figure S2 in Supplementary Material) (38). Furthermore, multiple sequence alignment of trout P2X_7_R with other known P2X_7_R sequences from representative vertebrates was performed using the CLUSTAL X version 1.8 and displayed by GeneDoc software (Figure S3 in Supplementary Material). The expression of P2X_7_R in the sorted IgM^+^, IgT^+^, and DN lymphocytes from peripheral blood and head kidney of healthy trout was performed by qPCR using the specific primer sets (Table 1) as described above. Moreover, the relative expression level of P2X_7_R in phagocytic and non-phagocytic trout B cells was determined as described in Section “Expression of AMP Genes in Phagocytic and Non-Phagocytic Trout B Cells.”

### Statistical Analysis

The statistic *p* value was calculated by one-way ANOVA with a Dunnett *post hoc* test (SPSS Statistics, version 19, IBM). A *p* value <0.05 was considered statistically significant.

## Results

### Constitutive Expression of AMP Genes in Trout B Cells

**Figure 1:**
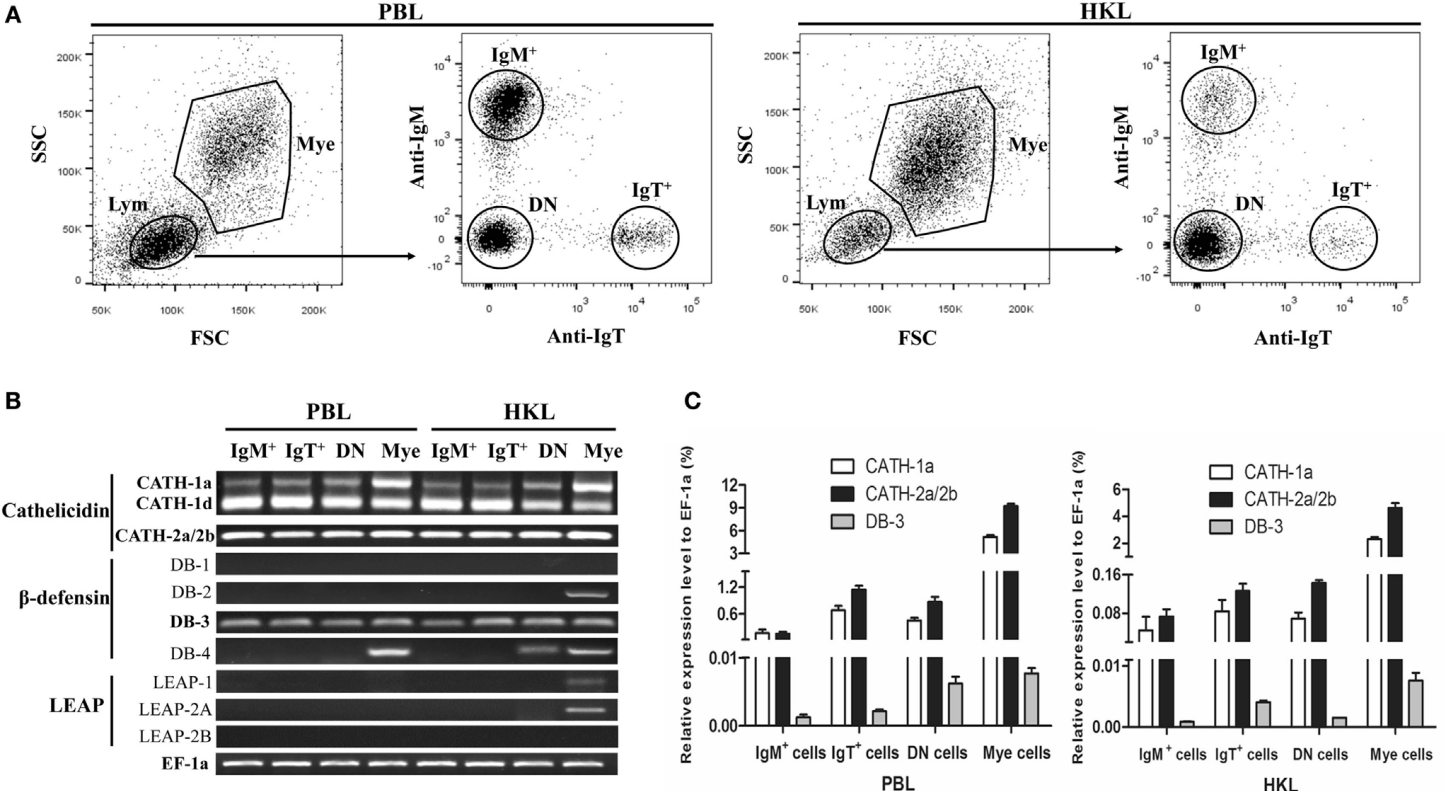
**Constitutive expression of antimicrobial peptide (AMP) genes in trout IgM^+^, IgT^+^, and DN lymphocytes as well as myeloid leukocytes**. **(A)** Flow cytometry of trout peripheral blood leukocytes (PBLs) and head kidney leukocytes (HKLs) double stained with mouse anti-trout IgM and anti-trout IgT monoclonal antibodies. Lymphocytes (Lym) and myeloid leukocytes (Mye) were gated, and the IgM^+^, IgT^+^, and IgM^−^IgT^−^ (DN) lymphocytes as well as myeloid leukocytes were FACS sorted and subjected to total RNA isolation and cDNA synthesis. **(B)** Expression patterns of AMP genes in trout IgM^+^, IgT^+^, and DN lymphocytes as well as myeloid leukocytes analyzed by normal RT-PCR. **(C)** Expression levels of AMP genes in trout IgM^+^, IgT^+^, and DN lymphocytes as well as myeloid leukocytes analyzed by quantitative real-time PCR and normalized against the expression of elongation factor 1a (EF-1a). Data are representative of three independent experiments [mean ± SD in panel **(C)**].

The expression patterns of AMP genes in the IgM^+^ and IgT^+^ B cells from trout peripheral blood and head kidney were analyzed by RT-PCR. So far, four cathelicidin, four β-defensin, and three LEAP genes have been discovered in trout. In the sorted IgM^+^ and IgT^+^ B cells (Figure 1A), the transcripts of all the four cathelicidin genes (CATH-1a, CATH-1d, CATH-2a, and CATH-2b) and only one β-defensin gene (DB-3) could be detected (Figure 1B). Since CATH-2a and CATH-2b are highly homologous genes (99% cDNA sequence identity), the PCR products were sequenced to verify the co-expression of both genes in trout IgM^+^ and IgT^+^ B cells, and the results showed that the expression level of CATH-2a was much higher than that of CATH-2b (data not shown). As expected, trout IgM^+^ and IgT^+^ B cells could not express LEAP genes, a family of AMP genes mainly expressed in the liver. However, the expression of the β-defensin genes DB-2 and DB-4 as well as the LEAP genes LEAP-1 and LEAP-2A could be detected in myeloid leukocytes and part of the DN lymphocytes. The expression levels of CATH-1a, CATH-2a/2b, and DB-3 in trout IgM^+^ and IgT^+^ B cells were further analyzed by qPCR and compared with those of DN lymphocytes and myeloid leukocytes. As shown in Figure 1C, the expression level of CATH-2a/2b in trout IgM^+^ and IgT^+^ B cells was the highest, followed by CATH-1a, while the expression level of DB-3 was the lowest. The expression levels of these AMP genes in the DN lymphocytes were generally similar to those in trout B cells. However, in myeloid leukocytes, the expression levels of these AMP genes were significantly higher than those of lymphocytes.

### Immunofluorescence Detection of Cathelicidin Peptide CATH-2a in Trout B Cells

**Figure 2:**
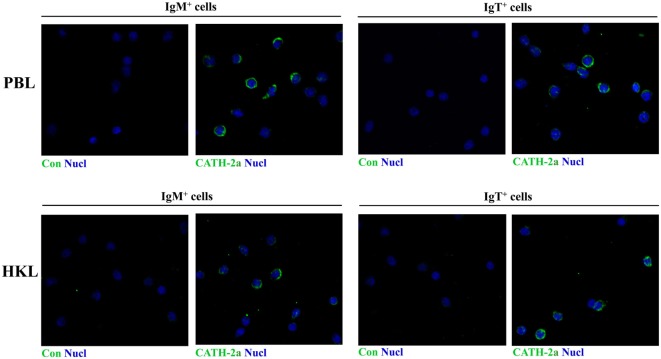
**Immunofluorescence detection of cathelicidin peptide CATH-2a in trout B cells**. The IgM^+^ and IgT^+^ B cells were MACS sorted from peripheral blood leukocytes (PBLs) and head kidney leukocytes (HKLs) of trout. Then cells were stained with rabbit anti-trout CATH-2a or control (rabbit anti-GST) Ab (green), and with DAPI for nuclei (blue). Data are representative of two independent experiments.

Immunofluorescence was performed to detect the expression of CATH-2a in the IgM^+^ and IgT^+^ B cells sorted from peripheral blood and head kidney of trout. As shown in Figure 2, the fluorescence signals of CATH-2a could be detected in most of the IgM^+^ and IgT^+^ B cells. The negative control showed no immunoreactive signals.

### Induced Expression of AMP Genes in Phagocytic Trout B Cells

**Figure 3:**
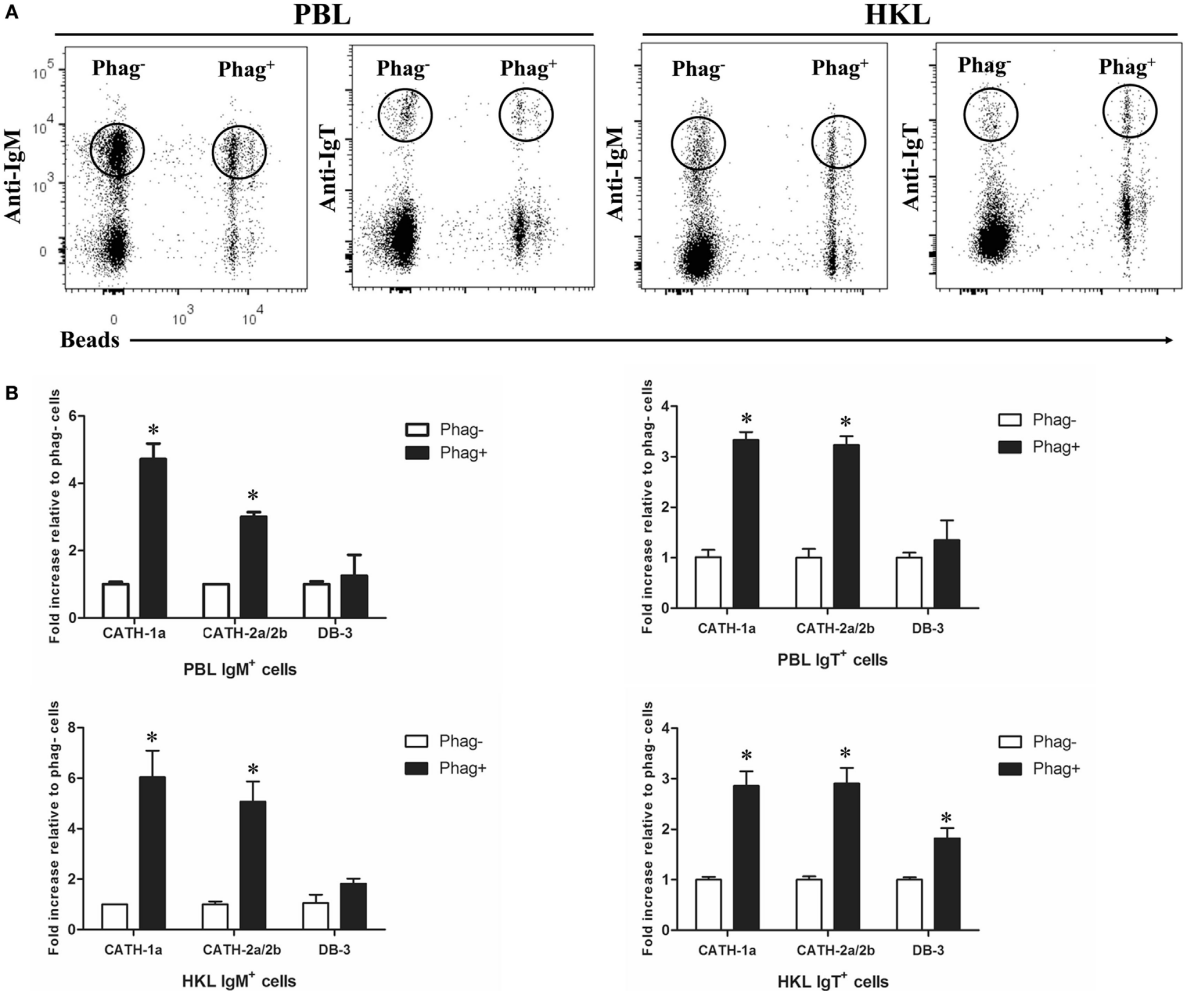
**Induced expression of antimicrobial peptide (AMP) genes in trout phagocytic B cells**. **(A)** Flow cytometry of trout peripheral blood leukocytes (PBLs) and head kidney leukocytes (HKLs) incubated with 1.0 µm fluorescent beads (labeled with FITC) for 3 h and double stained with mouse anti-trout IgM and anti-trout IgT monoclonal antibodies. Lymphocytes were gated, and the phagocytic and non-phagocytic IgM^+^ and IgT^+^ B cells were FACS sorted and subjected to total RNA isolation and cDNA synthesis. **(B)** The relative expression levels of AMP genes in the phagocytic and non-phagocytic trout B cells were determined by quantitative real-time PCR and normalized against the internal control elongation factor 1a. The *p* value was calculated by ANOVA with Dunnett *post hoc* test (*p < 0.05). Phag^−^, non-phagocytic cells; Phag^+^, phagocytic cells. Data are representative of three independent experiments [mean ± SD in panel **(B)**].

Previous studies have shown that trout IgM^+^ and IgT^+^ B cells have potent phagocytic activities and they form phagolysosomes after the internalization of particles to degrade the phagocytosed particles (7, 8). Thus, in order to determine if the AMPs in trout IgM^+^ and IgT^+^ B cells contribute to this intracellular degradation process, we compared the expression levels of AMP genes between the phagocytic and non-phagocytic B cells. As shown in Figure 3A, phagocytic and non-phagocytic IgM^+^ and IgT^+^ B cells from peripheral blood and head kidney of trout were sorted using fluorescent beads. Further study revealed that the expression of the cathelicidin peptide genes CATH-1a and CATH-2a/2b in phagocytic IgM^+^ and IgT^+^ B cells increased dramatically, when compared with non-phagocytic B cells. However, unlike the cathelicidin peptide genes, the expression of the β-defensin gene DB-3 in phagocytic and non-phagocytic IgM^+^ and IgT^+^ B cells did not change significantly (Figure 3B).

### Induced Expression of AMP Genes in Trout B Cells *In Vitro* and *In Vivo* by LPS or *A. salmonicida*

**Figure 4:**
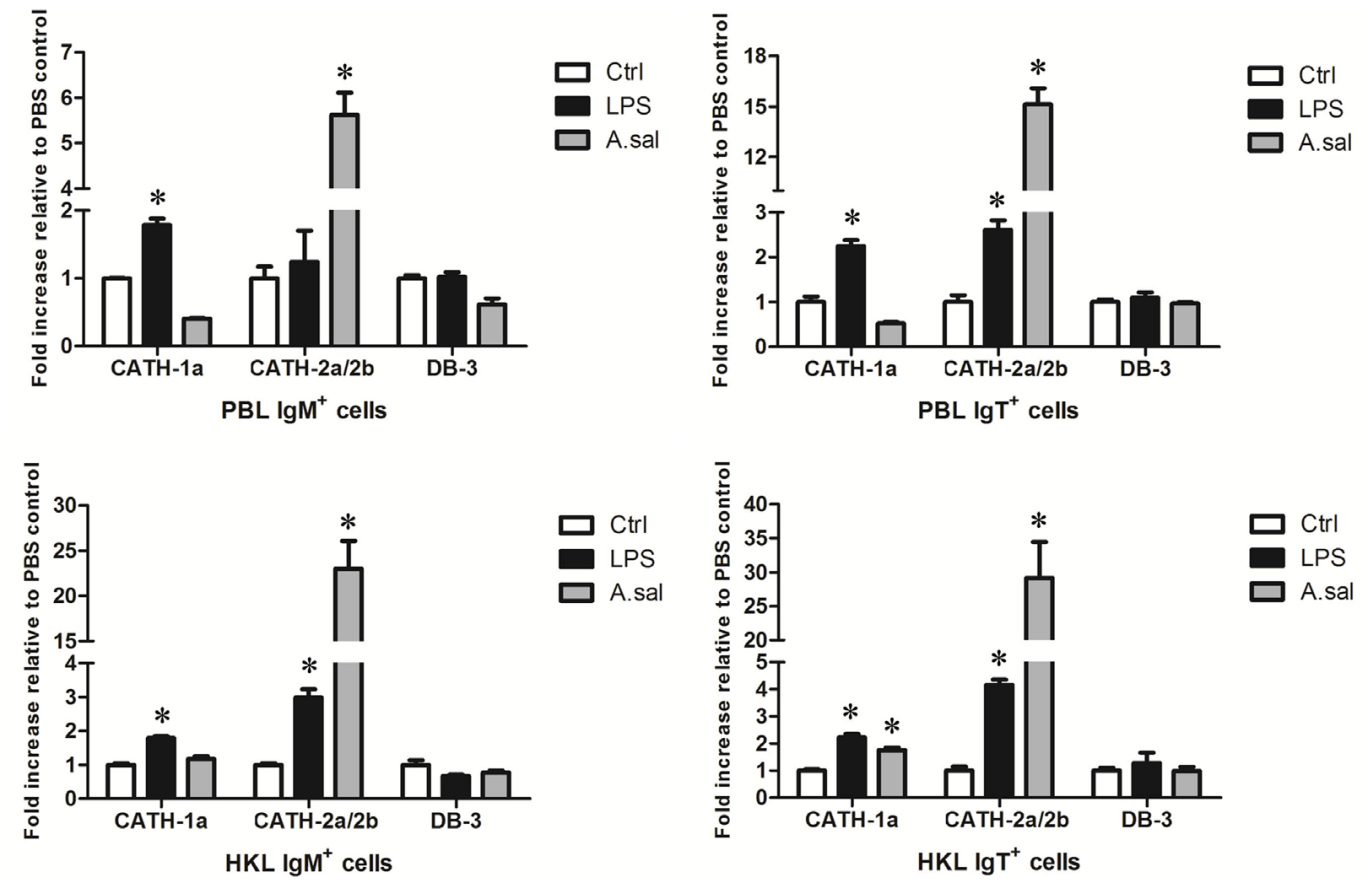
***In vitro*-induced expression of antimicrobial peptide (AMP) genes in trout B cells by LPS or *Aeromonas salmonicida***. The IgM^+^ and IgT^+^ B cells were MACS sorted from peripheral blood leukocytes (PBLs) and head kidney leukocytes (HKLs) of trout and incubated with PBS (Ctrl), LPS (25 µg/ml), or heat-killed *A. salmonicida* (A.sal; cell:bacteria = 1:10) for 8 h at 17°C. Then cells were collected and subjected to total RNA isolation and cDNA synthesis. The relative expression levels of AMP genes in trout IgM^+^ and IgT^+^ B cells under normal and challenged situations were determined by quantitative real-time PCR and normalized against elongation factor 1a (*p < 0.05). Data are representative of three independent experiments (mean ± SD).

LPS is the major component of the outer membrane of Gram-negative bacteria, and it can elicit strong immune responses in animals. To assess its potential role in the induction of AMP gene expression in trout B cells, trout IgM^+^ and IgT^+^ B cells from peripheral blood and head kidney were stimulated with LPS for 8 h. As shown in Figure 4, in general, the expression of the cathelicidin peptide genes CATH-1a and CATH-2a/2b, but not the β-defensin gene DB-3, in trout IgM^+^ and IgT^+^ B cells increased dramatically, with the fold increase of CATH-2a/2b higher than that of CATH-1a. As expected, the salmonid pathogenic bacterium *A. salmonicida* also significantly stimulated the expression of CATH-2a/2b in trout B cells, and the effect of stimulation was higher than that of LPS. However, at the time point tested, the expression of the CATH-1a and DB-3 in *A. salmonicida*-stimulated trout IgM^+^ and IgT^+^ B cells did not change significantly.

**Figure 5:**
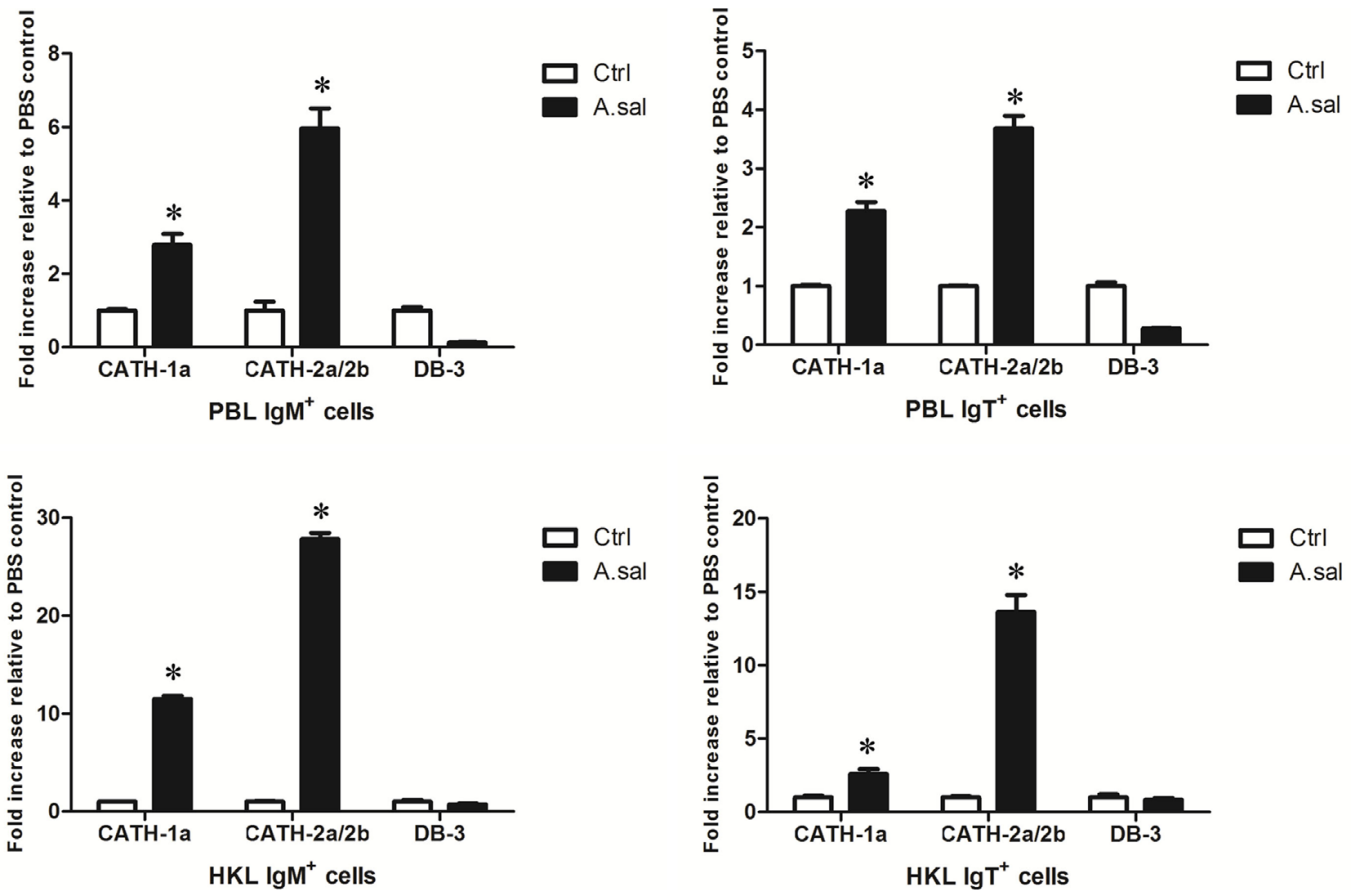
***In vivo*-induced expression of antimicrobial peptide (AMP) genes in trout B cells by *Aeromonas salmonicida***. Healthy trout were injected intraperitoneally with PBS (Ctrl) or *A. salmonicida* (A.sal). The IgM^+^ and IgT^+^ B cells were MACS sorted from peripheral blood leukocytes (PBLs) and head kidney leukocytes (HKLs) of trout at 30 h postinfection and then subjected to total RNA isolation and cDNA synthesis. The relative expression levels of AMP genes in the IgM^+^ and IgT^+^ B cells from healthy and infected trout were determined by quantitative real-time PCR and normalized against elongation factor 1a (*p < 0.05). Data are representative of three independent experiments (mean ± SD).

In addition to *in vitro* stimulations, *in vivo A. salmonicida* infection was conducted to detect whether AMPs in trout B cells participated in the early immune response of hosts to invading bacteria. As shown in Figure 5, *A. salmonicida* infection significantly provoked the expression of CATH-1a and CATH-2a/2b, especially CATH-2a/2b, in trout IgM^+^ and IgT^+^ B cells. However, the expression of DB-3 was not changed or was decreased in trout B cells after infection.

### Cathelicidin Peptides CATH-1a and CATH-2a Enhance the Phagocytic Activity of Trout B Cells

**Figure 6:**
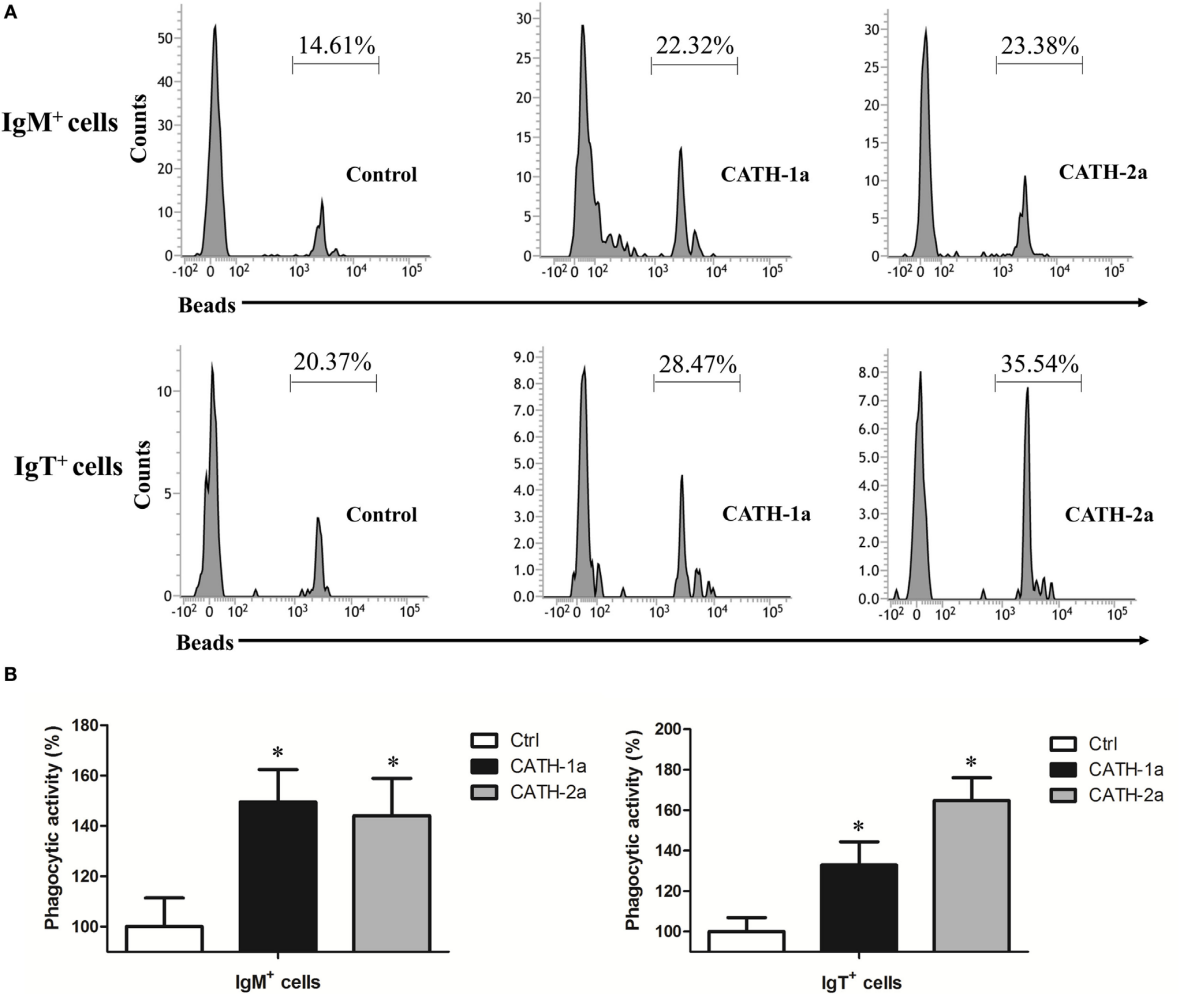
**Cathelicidin peptides enhance the phagocytic activity of trout B cells**. **(A)** Flow cytometry to detect the phagocytic activity of trout B cells stained with mouse anti-trout IgM and anti-trout IgT monoclonal antibodies (mAbs). **(B)** Trout CATH-1a and CATH-2a can enhance the phagocytic activity of B cells. Peripheral blood leukocytes were incubated for 3 h at 17°C with trout CATH-1a or CATH-2a (2 µM). Non-stimulation controls (Ctrl) were included, with PBS instead of peptide. Then cells were incubated with 1.0 µm fluorescent beads (labeled with FITC; cell:bead = 1:15) for 1 h at 17°C and double stained with mouse anti-trout IgM and anti-trout IgT mAbs. Finally, the phagocytic activity was measured by flow cytometry (*p < 0.05). Data are representative of three independent experiments [mean ± SD in panel **(B)**].

Since a previous study has shown that cathelicidin peptide LL-37 could increase the phagocytic activity of human macrophages (24), we determined if cathelicidin peptides could also modulate this activity of fish B cells. As shown in Figure 6, both CATH-1a and CATH-2a significantly increased the phagocytic activity of trout IgM^+^ and IgT^+^ B cells in peripheral blood. CATH-1a increased the phagocytic activity of IgM^+^ and IgT^+^ B cells to 149.4 and 132.9%, respectively. Meanwhile, CATH-2a increased the phagocytic activity of IgM^+^ and IgT^+^ B cells to 144.1 and 164.8%, respectively. Since our data could not completely differentiate the beads that were internalized from those that were cell surface bound, the actual stimulating activities of CATH-1a and CATH-2a might be higher than the calculated values.

### Cathelicidin Peptides CATH-1a and CATH-2a Enhance the Intracellular Bactericidal Activity of Trout B Cells

**Figure 7:**
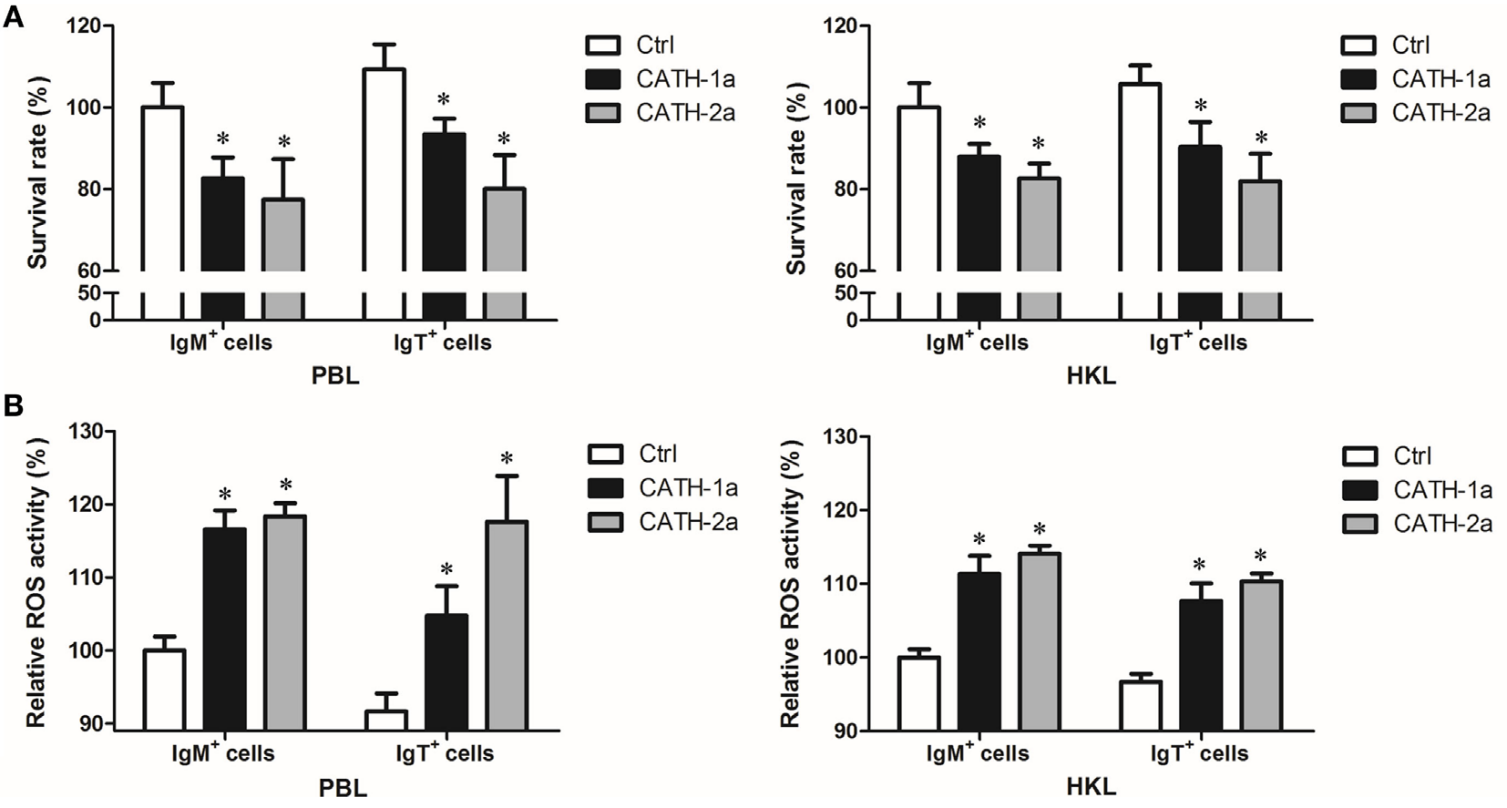
**Cathelicidin peptides enhance the intracellular bactericidal and reactive oxygen species (ROS) activities of trout B cells**. **(A)** Cathelicidin peptides can enhance the intracellular bactericidal activities of trout B cells. Peripheral blood leukocytes (PBLs) and head kidney leukocytes (HKLs) of trout were incubated with *Escherichia coli* for 4 h at 17°C and then stained with mouse anti-trout IgM or anti-trout IgT mAb. The IgM^+^ and IgT^+^ B cells were MACS sorted and incubated with CATH-1a or CATH-2a (2 µM) for 3 h at 17°C. Non-stimulation controls (Ctrl) were included, with PBS instead of peptide. After incubation, cells were washed, lysed, and plated onto LB agar plates. Viable bacteria were counted after incubation at 37°C overnight, and results are presented as percentage of the number of bacteria for stimulation group/control group. The survival rate of the control group of IgM^+^ B cells was set as 100%. **(B)** Cathelicidin peptides can enhance the intracellular ROS activities of trout B cells. Above MACS-sorted IgM^+^ and IgT^+^ B cells from PBLs and HKLs of trout were incubated with CATH-1a or CATH-2a (2 µM) for 3 h at 17°C. Non-stimulation controls (Ctrl) were included, with PBS instead of peptide. After incubation, cells were stained with 2,7-dichlorofluorescin diacetate for 1 h at 17°C. The fluorescence intensity of the cells was read using a fluorescence microplate reader, and the relative ROS activity was calculated. The ROS activity of the control group of IgM^+^ B cells was set as 100% (*p < 0.05). Data are representative of three independent experiments (mean ± SD).

Since a previous study has shown that cathelicidin peptide LL-37 could increase the intracellular bactericidal activity of human macrophages (25), it was worth determining if cathelicidin peptides could also increase this activity of fish B cells. As shown in Figure 7A, our results demonstrated that the intracellular bactericidal activity of trout IgM^+^ and IgT^+^ B cells was significantly increased after treatment with CATH-1a or CATH-2a. In addition, the stimulating effect of CATH-2a was higher than that of CATH-1a. For peripheral blood-derived B cells, CATH-1a decreased the survival rate of intracellular bacteria ingested by IgM^+^ and IgT^+^ B cells to 82.6 and 85.5% of each control, respectively, while CATH-2a decreased the survival rate to 77.4 and 73.2%, respectively. For head kidney-derived B cells, the capacities of CATH-1a and CATH-2a to enhance the intracellular bactericidal activity were slightly weaker than those of peripheral blood-derived B cells.

### Cathelicidin Peptides CATH-1a and CATH-2a Enhance the Intracellular ROS Activity of Trout B Cells

In order to investigate the potential mechanisms for how cathelicidin peptides contributed to the intracellular bactericidal activity of trout B cells, we tested the intracellular ROS activity of *E. coli*-phagocytosed IgM^+^ and IgT^+^ B cells treated with CATH-1a and CATH-2a. As shown in Figure 7B, compared with the control cells, significantly increased ROS activity in CATH-1a- or CATH-2a-treated trout IgM^+^ and IgT^+^ B cells was found. In peripheral blood-derived IgM^+^ and IgT^+^ B cells, CATH-1a increased the ROS activity to 116.6 and 114.3% of each control, respectively, while CATH-2a increased the activity to 118.3 and 128.3%, respectively. In head kidney-derived trout B cells, the cathelicidin peptides showed a slightly weaker capacity to increase the ROS activity than that in the peripheral blood-derived B cells. In general, the ROS-inducing activity of CATH-2a was slightly higher than that of CATH-1a.

### Expression of P2X_7_R in Trout B Cells

**Figure 8:**
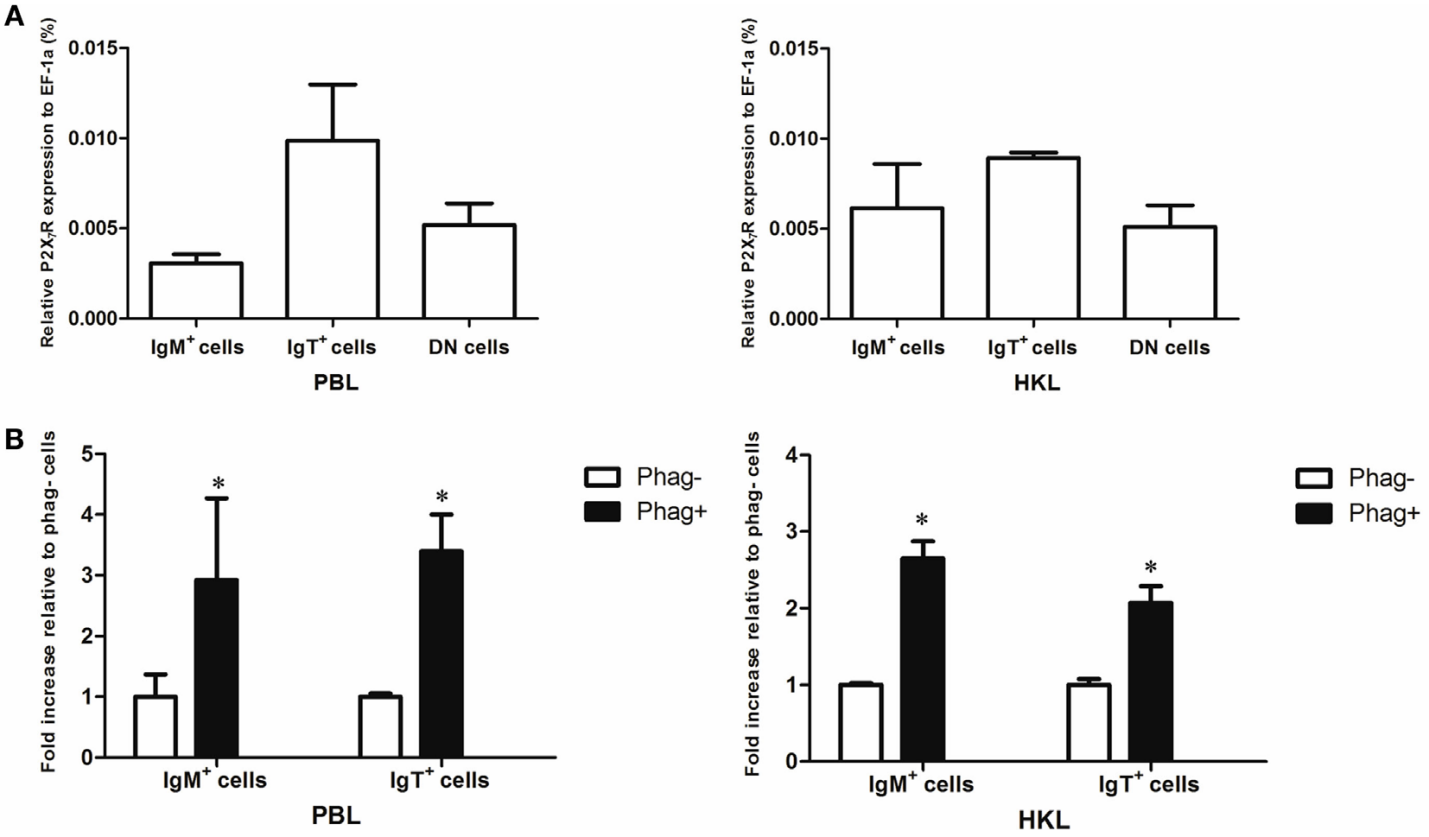
**Expression of P2X_7_ receptor (P2X_7_R) in normal and phagocytic trout B cells**. **(A)** Expression level of P2X_7_R in normal trout B cells. The expression level of P2X_7_R in FACS-sorted trout IgM^+^, IgT^+^, and IgM^−^IgT^−^ (DN) lymphocytes from peripheral blood leukocytes (PBLs) and head kidney leukocytes (HKLs) was analyzed by quantitative real-time PCR (qPCR) and normalized against the expression of elongation factor 1a (EF-1a). **(B)** Induced expression of P2X_7_R in the phagocytic IgM^+^ and IgT^+^ B cells, which were FACS sorted from PBLs and HKLs of trout. The relative expression level of P2X_7_R in the phagocytic and non-phagocytic IgM^+^ and IgT^+^ B cells of trout was determined by qPCR and normalized against EF-1a (*p < 0.05). Phag^−^, non-phagocytic cells; Phag^+^, phagocytic cells. Data are representative of three independent experiments (mean ± SD).

Since previous studies have shown that cathelicidin peptide could enhance the phagocytic, bactericidal, and ROS activities of human and fish macrophages through P2X_7_R (25, 39), we determined if cathelicidin peptides could also modulate these activities of trout B cells through this receptor. Trout P2X_7_R was cloned (Figures S2 and S3 in Supplementary Material), and its expression in IgM^+^ and IgT^+^ B cells was analyzed by qPCR (Figure 8). As expected, trout IgM^+^ and IgT^+^ B cells could express P2X_7_R, with the expression level in IgT^+^ B cells higher than that in IgM^+^ B cells and DN lymphocytes (Figure 8A). Moreover, the expression of P2X_7_R in the phagocytic IgM^+^ and IgT^+^ B cells increased dramatically when compared with the non-phagocytic B cells (Figure 8B).

## Discussion

Over the past decade, researchers have achieved considerable progress in the immune functions of fish B cells, and fish B cells have been proven to play multiple roles in innate immunity (7–9, 12). This inspired us to further explore the innate immune functions of fish B cells. Since AMPs represent an evolutionarily old component of the innate immune system of animals, it was assumed that investigating the interactions between fish B cells and AMPs would provide novel insights into the innate nature of B cells in lower vertebrates.

Previous studies have shown that human B cells can produce cathelicidin peptide LL-37, α-defensins HNP 1–3, and β-defensin 2 (18, 21). Mouse B cells can also produce cathelicidin peptide mCRAMP (20). In this study, trout IgM^+^ and IgT^+^ B cells also expressed multiple AMP genes, including four cathelicidin peptide genes and one β-defensin gene. Further study revealed that the expression levels of the cathelicidin peptide genes CATH-1a and CATH-2a/2b in the phagocytic trout B cells were higher than those in the non-phagocytic B cells, indicating that intracellular particles can generate upregulating signals of AMPs in fish B cells. Moreover, *in vitro* LPS and *A. salmonicida* stimulations also increased the expression of the cathelicidin peptide genes, especially CATH-2a/2b, in trout B cells, indicating that fish B cells can recognize bacterial stimulants through pattern recognition receptors and generate upregulating signals of AMPs. Most importantly, *in vivo A. salmonicida* infection significantly provoked the expression of the cathelicidin peptide genes, especially CATH-2a/2b, in trout B cells, indicating that AMPs in fish B cells participate in the early immune response of hosts to invading bacteria. Since the B cells used in the *in vitro* and *in vivo* stimulations were sorted by MACS, which might be contaminated by some non-B leukocytes, we cannot rule out that part of the upregulated gene expression of B cells was affected by the putative contaminated myeloid cells. Unlike the cathelicidin peptide genes, the expression of the β-defensin gene DB-3 in trout B cells did not respond to the intracellular, extracellular, *in vitro*, and *in vivo* stimulations, indicating that β-defensin is not the main innate immune effector of fish B cells.

Studies have proven that AMPs in mammals are mainly produced by neutrophils (18, 19). Moreover, monocytes/macrophages and lymphocytes can also produce AMPs (18, 20). However, to our knowledge, no research study has compared the AMP gene expression levels of myeloid leukocytes and lymphocytes, not to mention B cells, in lower vertebrates. In this study, we found that, similar to mammals, trout AMP genes were also mainly expressed by myeloid leukocytes. Moreover, the AMP gene expression patterns and levels of B cells and other lymphocytes were generally similar, except for the β-defensin gene DB-4.

In addition to their direct antimicrobial effects, AMPs possess other modulatory activities, with their expression activating more complex immune responses (22). Previous studies have shown that cathelicidin peptide LL-37 can enhance the phagocytic, intracellular bactericidal, ROS, and lysosome formation activities of human macrophages (24, 25). Moreover, a β-defensin in Atlantic cod can enhance the phagocytic activity of attached HKLs (26). Unlike mammalian B cells, fish B cells possess potent innate immune activities like macrophages (7). Thus, we have reasons to speculate that fish AMPs can act on B cells, like human AMPs acting on macrophages. As expected, the results in this study confirmed the hypothesis. Cathelicidin peptides CATH-1a and CATH-2a both could enhance the phagocytic, intracellular bactericidal, and ROS activities of trout B cells, with the stimulating effect of CATH-2a higher than that of CATH-1a. Moreover, IgM^+^ and IgT^+^ B cells were sorted by MACS from trout peripheral blood and head kidney and were stimulated directly with CATH-1a and CATH-2a. The results showed that cathelicidin peptides could also increase the ROS activities in trout B cells that have not phagocytosed (data not shown). Since the B cells used in the intracellular bactericidal and cellular ROS assays were obtained by MACS, which might contain some non-B leukocytes, we cannot exclude the possibility that part of the stimulating effects of B cells was affected by the portion of contaminated myeloid cells. Although a recent study has shown that CK9, a CCL25-like chemokine, can upregulate the phagocytic capacity of trout IgM^+^ B cells but has no effect on the IgT^+^ B cells (37), it is the first time that the current study describes two innate immune molecules that can upregulate the phagocytic capacities of both IgM^+^ and IgT^+^ B cells in fish. Previous studies have proven that IgT^+^ B cells are the main B-cell lineage specialized in mucosal immunity (8, 27, 28, 40). This makes the stimulating effect of cathelicidin peptides on fish IgT^+^ B cells particularly important, when encountering invading pathogens at mucosal surfaces. The potential application of cathelicidin peptides as immune enhancers in aquaculture is worth further research. Previous studies have proven that human and mouse β-defensin peptides can modulate the immune activities of macrophages, immature dendritic cells, and CD4^+^ T cells (41–43). Unfortunately, due to the multiple cysteines in the molecule, we failed to synthesize trout β-defensin peptide DB-3. Whether β-defensin peptides can modulate the immune activities of fish B cells deserves further study.

Previous studies have proven that B cells from early vertebrates have potent phagocytic and microbicidal abilities like macrophages (7). Moreover, bipotential progenitors of B cells and macrophages were found in murine fetal liver and adult bone marrow (44, 45). These evidences indicate that vertebrate B cells could have evolved from macrophages or ancient phagocytic cells. In this study, we found that the immune activities of fish B cells can be modulated by AMPs, which has been proven in human and fish macrophages (24, 25, 39). This not only helps to fully understand the innate nature of B cells in fish but also provides new evidence for understanding the close relationship between B cells and macrophages in vertebrates.

P2X_7_ receptor is an ATP-gated ion channel, which plays an important role in the innate immune response of animals (46). In human, P2X_7_R has been proven to mediate the stimulating effects of LL-37 on the bactericidal, ROS, and lysosome formation activities of macrophages (25). In ayu (*Plecoglossus altivelis*), knockdown of P2X_7_R significantly reduced the stimulating effects of cathelicidin peptide CATH on the phagocytic, bactericidal, and ROS activities of monocytes/macrophages (39, 47). In this study, we found that trout B cells could also express P2X_7_R, with the expression level in the IgT^+^ B cells higher than that in the IgM^+^ B cells. In addition, we found that the expression level of trout P2X_7_R in phagocytic IgM^+^ and IgT^+^ B cells was significantly higher than that of non-phagocytic B cells, indicating that P2X_7_R might play a role in the phagocytosis of fish B cells, which has been proven in fish macrophages (47). However, whether the immunomodulatory effect of fish cathelicidin peptides on B cells is also mediated by P2X_7_R needs to be further clarified.

It is worth noting that trout B cells constitute about 57, 55, 47, and 20% of all lymphocytes in the blood, spleen, peritoneal cavity, and head kidney, respectively (8), much higher than the percentages in humans. Considering the large number of B cells in trout, the expression of multiple AMP genes in trout B cells can be significantly upregulated in response to stimulations, and the immune activities of B cells can be modulated by AMPs, these results collectively suggest that B cells play important roles in the innate immunity of fish.

## Ethics Statement

All animal experiments were approved by the Committee on the Ethics of Animal Experiments of the Institute of Hydrobiology, Chinese Academy of Sciences.

## Author Contributions

X-JZ performed most of the experiments, analyzed the data, and wrote the manuscript. PW helped with most of the experiments. NZ helped with cell sorting. D-DC helped with reagent preparation and data analysis. PN helped with experiment design. J-LL and Y-AZ designed the research and revised the manuscript.

## Conflict of Interest Statement

The authors declare that the research was conducted in the absence of any commercial or financial relationships that could be construed as a potential conflict of interest. Before accepting the reviewer position, the reviewer, OS, declared a past supervisory role with one of the authors, Y-AZ, to the handling Editor, who ensured that the process met the standards of a fair and objective review.

## Footnotes

^1^http://www.ncbi.nlm.nih.gov.

^2^http://www.ncbi.nlm.nih.gov/genbank/.
