# Supplementary material for: B Cell Functions Can Be Modulated by Antimicrobial Peptides in Rainbow Trout Oncorhynchus mykiss: Novel Insights into the Innate Nature of B Cells in Fish
Source: Front Immunol. 2017 Apr 4;8:388. doi: 10.3389/fimmu.2017.00388 (PMC5378723; doi:10.3389/fimmu.2017.00388)
Supplement: Supplementary file 1 [file Data_Sheet_1.pdf]

Supplementary Material

B Cell Functions Can Be Modulated by Antimicrobial Peptides in Rainbow Trout *Oncorhynchus mykiss*: Novel Insights into the Innate Nature of B cells in Fish

Xu-Jie Zhang, Peng Wang, Nu Zhang, Dan-Dan Chen, Pin Nie, Jia-Le Li*, and Yong-An Zhang*

*** Correspondence:** Yong-An Zhang: yzhang@ihb.ac.cn; Jia-Le Li: jlli@shou.edu.cn

Supplementary Figures

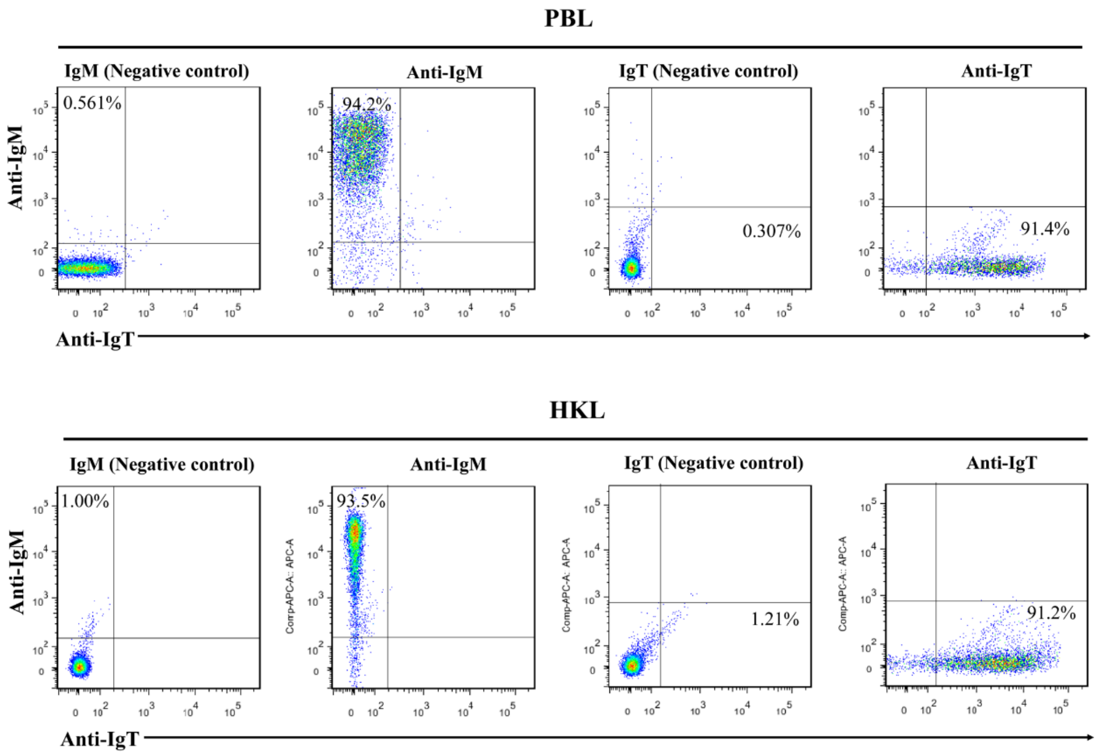

Supplementary Figure 1 | Assessment of the purity of trout B cells sorted by MACS. Trout IgM^+ and IgT^+ B cells were sorted by magnetic-activated cell sorting (MACS) from the peripheral blood leukocytes (PBL) and head kidney leukocytes (HKL) with LS separation columns and double stained with mouse anti-trout IgM and anti-trout IgT mAbs. The purity of the sorted cells was determined by flow cytometry. The number in each quadrant indicates the percentage of cells.

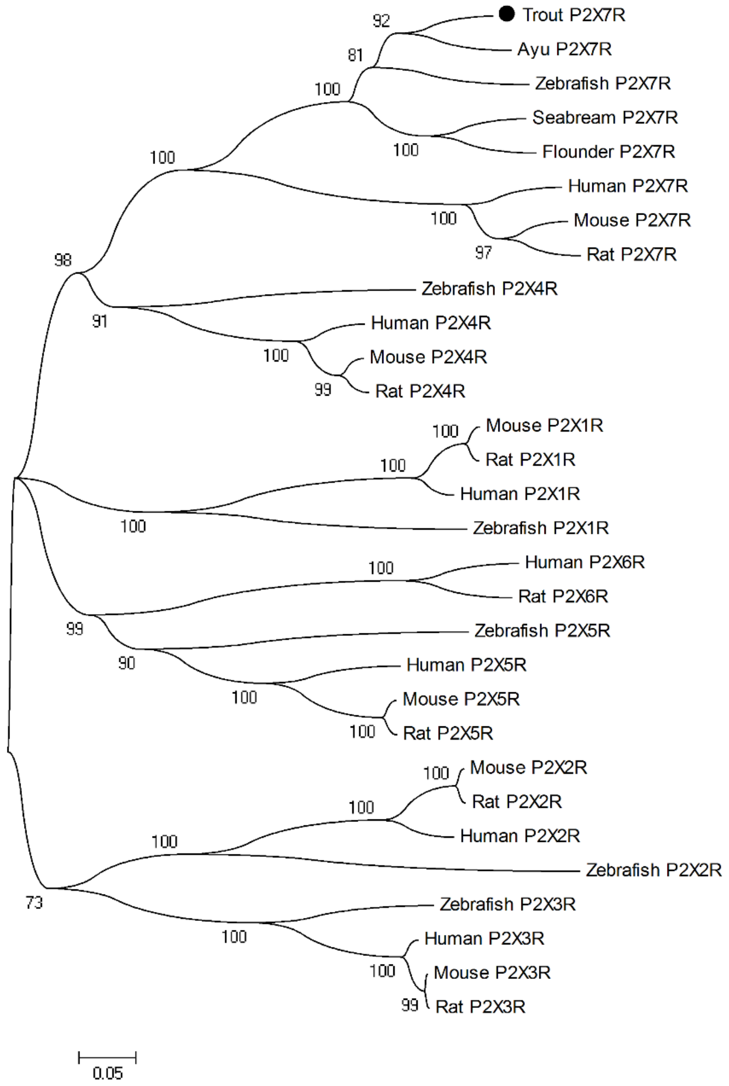

Supplementary Figure 2 | Phylogenetic relationship of trout $P2X_7R$ with selected vertebrate P2X receptor family members. Neighbor-joining phylogenetic tree was generated based on the amino acid sequence alignment using MEGA 4.1 package with 1000 bootstrap replications. GenBank accession numbers of the P2X receptor members are shown below: Human $P2X_1R$ (AAC24494.1), Human $P2X_2R$ (NP_733782.1), Human $P2X_3R$ (NP_002550.2), Human $P2X_4R$ (NP_001243725.1), Human $P2X_5R$ (NP_002552.2), Human $P2X_6R$ (AAF13303.1), Human $P2X_7R$ (NP_002553), Mouse $P2X_1R$ (NP_032797.3), Mouse $P2X_2R$ (AAK95327.2), Mouse $P2X_3R$ (NP_663501.2), Mouse $P2X_4R$ (NP_035156.2), Mouse $P2X_5R$ (NP_201578.2), Mouse $P2X_7R$ (CAD33539), Rat $P2X_1R$ (NP_037129.1), Rat $P2X_2R$ (NP_446108.2), Rat $P2X_3R$ (NP_112337.2), Rat $P2X_4R$ (NP_113782.1), Rat $P2X_5R$ (NP_542958.2), Rat $P2X_6R$ (CAA66044.1), Rat $P2X_7R$ (NP_062129), Zebrafish $P2X_1R$ (NP_945333.1), Zebrafish $P2X_2R$ (NP_945334.1), Zebrafish $P2X_3R$ (NP_945337.2), Zebrafish $P2X_4R$ (NP_705939.1), Zebrafish $P2X_5R$ (NP_919394.1), Zebrafish $P2X_7R$ (NP_945335), Seabream $P2X_7R$ (CAI59608.1), Flounder $P2X_7R$ (KC748421), Ayu $P2X_7R$ (CCM43769.1), and Trout $P2X_7R$ (KY088056).

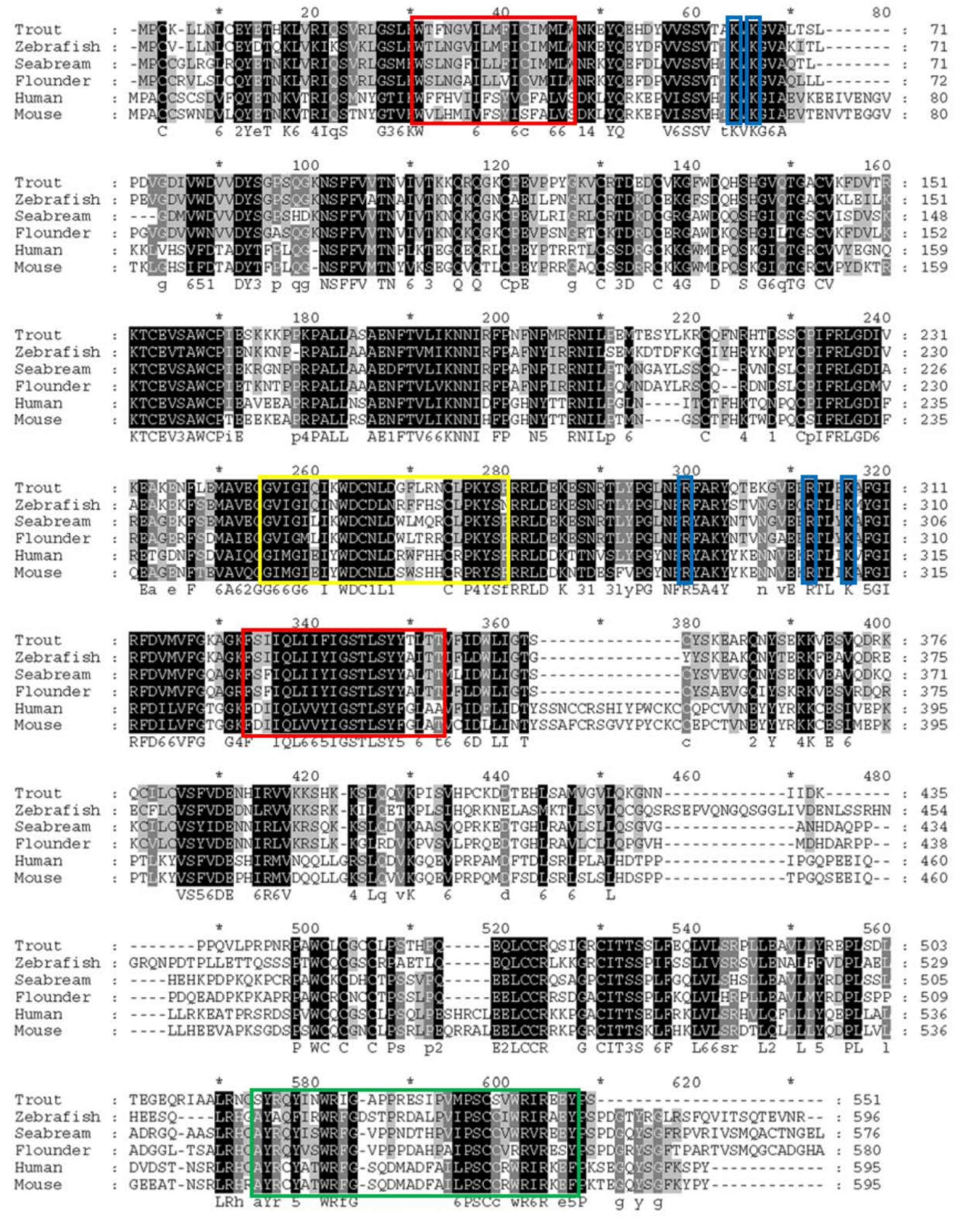

Supplementary Figure 3 | Multiple sequence alignment of trout P2X$_7$R with other representative vertebrate P2X$_7$R. Similar or identical amino acid residues are shadowed gray or black. Two transmembrane domains (red), five important residues for nucleotide binding (blue), the P2X family signature motif (yellow), and the LPS/lipid-binding domain (green) are boxed. GenBank accession numbers of the P2X$_7$R are shown below: Human P2X$_7$R (NP_002553), Mouse P2X$_7$R (CAD33539), Zebrafish P2X$_7$R (NP_945335), Seabream P2X$_7$R (CAI59608.1), Flounder P2X$_7$R (KC748421), and Trout P2X$_7$R (KY088056).
